# Supplementary material for: The interactome of intact mitochondria by cross-linking mass spectrometry provides evidence for coexisting respiratory supercomplexes
Source: Mol Cell Proteomics. 2017 Dec 8;17(2):216–32. doi: 10.1074/mcp.RA117.000470 (PMC5795388; doi:10.1074/mcp.RA117.000470)
Supplement: Supplemental Data [file supp_RA117.000470_133922_0_supp_23978_fzffwf.zip › spectra_annotation/mito_DR_spectra_annotation/469-1-8-1-11-1.pdf]

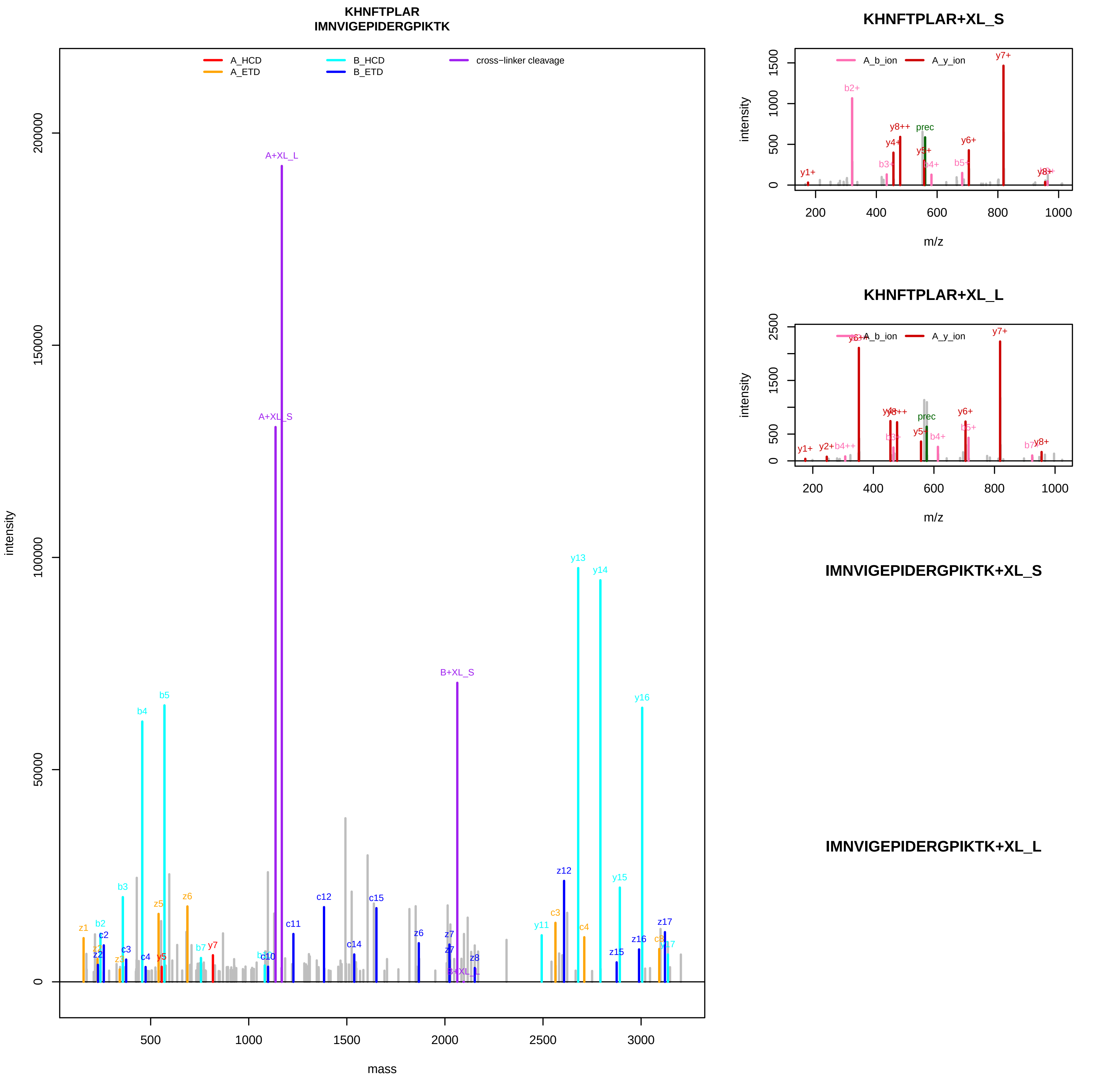

KHNFTPLAR
IMNVIGEPIDERGPIKTK
A_HCD
A_ETD
B_HCD
B_ETD
cross−linker cleavage
A+XL_L
A+XL_S
B+XL_S
B+XL_L
intensity
mass
0
50000
100000
150000
200000
500
1000
1500
2000
2500
3000
y13
y14
y16
y15
y11
y17
z12
z17
z16
z15
c3
c4
c8
c11
c12
c14
c15
c10
z6
z7
z8
b2
b3
b4
b5
b7
c2
c3
c4
z1
z2
z3
z5
z6
y5
y7
KHNFTPLAR+XL_S
KHNFTPLAR+XL_L
A_b_ion
A_y_ion
m/z
intensity
prec
y1+
y2+
y4+
y5+
y6+
y7+
y8+
y8++
y6++
b2+
b3+
b4+
b4++
b5+
b7+
200
400
600
800
1000
0
500
1000
1500
2500
IMNVIGEPIDERGPIKTK+XL_S
IMNVIGEPIDERGPIKTK+XL_L
